# Congenital cataract, facial dysmorphism and demyelinating neuropathy (CCFDN) in 10 Czech gypsy children – frequent and underestimated cause of disability among Czech gypsies

**DOI:** 10.1186/1750-1172-9-46

**Published:** 2014-04-01

**Authors:** Petra Lassuthova, Dana Šišková, Jana Haberlová, Iva Sakmaryová, Aleš Filouš, Pavel Seeman

**Affiliations:** 1 Department of Paediatric Neurology, DNA Laboratory 2nd Faculty of Medicine, Charles University in Prague and University Hospital Motol, Prague, Czech Republic; 2 Department of Paediatric Neurology, Thomayer’s Hospital, Prague, Czech Republic; 3 Department of Ophthalmology, 2nd Faculty of Medicine, Charles University in Prague and University Hospital Motol, Prague, Czech Republic

**Keywords:** Gypsies/genetics, Cataract/congenital, Nervous system diseases/*genetics, Facial nerve diseases/*congenital/genetics, Face/*abnormalities

## Abstract

**Background:**

Congenital Cataract Facial Dysmorphism and demyelinating Neuropathy (CCFDN, OMIM 604468) is an autosomal recessive multi-system disorder which was first described in Bulgarian Gypsies in 1999. It is caused by the homozygous founder mutation c.863 + 389C > T in the *CTDP1* gene. The syndrome has been described exclusively in patients of Gypsy ancestry. The prevalence of this disorder in the Gypsy population in the Czech Republic and Central Europe is not known and is probably underestimated and under-diagnosed.

**Methods:**

We clinically diagnosed and assessed 10 CCFDN children living in the Czech Republic. All patients are children of different ages, all of Gypsy origin born in the Czech Republic. Molecular genetic testing for the founder *CTDP1* gene mutation was performed.

**Results:**

All patients are homozygous for the c.863 + 389C > T mutation in the *CTDP1* gene.

All patients presented a bilateral congenital cataract and microphthalmos and had early cataract surgery. Correct diagnosis was not made until the age of two. All patients had variably delayed motor milestones. Gait is characteristically paleocerebellar in all the patients. Mental retardation was variable and usually mild.

**Conclusions:**

Clinical diagnosis of CCFDN should be easy for an informed pediatrician or neurologist by the obligate signalling trias of congenital bilateral cataract, developmental delay and later demyelinating neuropathy. Our data indicate a probably high prevalence of CCFDN in the Czech Gypsy ethnic subpopulation.

## Background

Congenital Cataract Facial Dysmorphism and demyelinating Neuropathy (CCFDN) (OMIM 604168) syndrome is a rare, complex developmental disorder with autosomal recessive inheritance. It is characterized by the obligate signalling trias of bilateral congenital cataract, developmental delay and later demyelinating neuropathy [1]. Congenital bilateral cataract is the first and invariable presenting sign [2]. Other ocular manifestations are also possible such as microcornea, microphthalmia, micropupil and floppy eyelid [3]. Symmetric demyelinating peripheral neuropathy with predominantly motor involvement develops later in life [4]. Mild facial dysmorphism develops in late childhood [5]. Additional features include: small stature, mild hypogonadism, mild cognitive deficit, cerebral and spinal cord atrophy on neuroimaging, occasional post-infectious rhabdomyolysis and osteoporosis [5,6].

The disorder is caused by the homozygous founder mutation c.863 + 389C > T in the *CTDP1* gene (the mutation is also known as IVS6 + 389C > T). This intronic mutation results in activation of an upstream cryptic splice acceptor site and causes aberrant splicing insertion of 95 nucleotides of the Alu sequence in the processed CTDP1 mRNA [7]. This mechanism had been identified previously only in ornithine aminotransferase deficiency (http://omim.org/entry/258870#0023). The insertion in the CTDP1 mRNA results in a premature termination signal 17 codons downstream of exon 6, with the mutant transcript expected to undergo nonsense-mediated decay or leads to a non-functional protein lacking the nuclear localization signal. Varon et al. [7] observed an abnormal product in all cell types studied, regardless of their involvement in the clinical phenotype which results in a premature stop codon. The syndrome has been described exclusively in patients of Gypsy ancestry [2,7]. An original description from 1999 reported 50 patients from 19 families from Bulgaria [2]. To date, there have only been a few patients reported from outside of Bulgaria, usually as single case reports. The prevalence of this disorder in the Gypsy population in the Czech Republic and Central Europe is not known with any accuracy and is probably underestimated and under-diagnosed.

In this article we use both appellations (Gypsies and Roma) as synonyms, as a name for this clearly defined and distinctive ethnic community, whose ancestors had migrated from the Indian subcontinent to Europe more than a millennium ago [8]. The current size of the European Gypsy population is estimated to be about eight million [9]. There were several Gypsy migration waves to Europe and accordingly, different subgroups exist today.

In the Czech Republic, a separate population of Bohemian Gypsies existed prior to the Second World War (WW II) but was almost exterminated in Nazi concentration camps. After WWII, a wave of Gypsies from Slovakia migrated to the Czech Republic. This wave was composed primarily of Romungro Gypsies which is the predominant sub-group in Slovakia and Hungary accounting for about 80% of the local populations. Also called Hungarian Gypsies, this sub-group migrated to Hungary about 650 years ago. A further sub-group, the Vlax Gypsies, arrived in the last century in Hungary and Slovakia, with a small number arriving in the Czech Republic.

The Gypsies represent genetically isolated subpopulations that arose from a limited number of founders. They therefore carry specific ethnic mutations and specific diseases. Some of the diseases are caused by mutations that originated in India. These diseases are common to the whole Gypsy population. Sharing of mutations and high carrier rates support a strong founder effect, and the identity of the congenital myasthenia 1267delG mutation in Gypsy and Indian/Pakistani chromosomes provided the best evidence yet of the Indian origins of the Gypsies [10]. Some of the other diseases are caused by mutations that arose later during the process of migration (these diseases are more or less specific for specific groups within the Gypsy population).

This type of mutational specificity has considerable implications and even advantages for the diagnostic DNA testing. The fact of genetic homogeneity among Gypsies simplifies diagnosis and prevention of these “private Gypsy” diseases, reducing costs. The general health among European Gypsies is worse than in the majority of populations in Central and South-Eastern Europe, with an associated lower life expectancy. High levels of both communicable and non-communicable diseases exist within Gypsy populations [11,12]. It is essential to try to improve this situation, increase awareness of the issue and to reduce their disadvantaged circumstances.

We describe a cohort of 10 CCFDN pediatric patients of different ages. Diagnosis of CCFDN should be easy for an informed pediatrician. Our data indicate a high prevalence of this syndrome among the Czech Gypsy population. The situation might be similar in other European countries.

## Methods

### Patients

We present ten non-related children of different ages with CCFDN. Patients were examined clinically (PS, JH or DS), electrophysiologically (JH and DS) and ophthalmologically (AF). All patients, or in the case of children, their parents, signed informed consent with the study which was approved by the ethics committee of University Hospital Motol.

### Controls

One hundred and sixteen control DNA samples from individuals of Gypsy origin were tested for the presence of the c.863 + 389C > T mutation. These 116 samples are anonymized and were provided by three human genetics departments – two from the Czech Republic (Ústí nad Labem and Brno – cities which are distant to each other) and one from Slovakia (Bratislava). These regions are all distant from one another.

### Methods

#### DNA sequence analysis

DNA was isolated from peripheral blood. Intron six of the *CTPD1* gene with the site of the c.863 + 389C > T mutation was amplified by PCR using intronic primers (sequences are available on request), directly sequenced using the same primers and the BigDye Terminator Ver. 3.1 sequencing kit (Applied Biosystems, Foster City, CA, USA), and analyzed on an ABI PRISM® 3100 Avant Genetic Analyzer (Applied Biosystems, Foster City, CA, USA). The obtained sequences were compared to a reference sequence NM_004715.4.

Analysis with restriction enzymes from a new PCR amplification was performed to confirm the mutation. For restriction analysis enzyme Nla III was used. This enzyme cuts the wild type sequence, while the mutant sequence is not cut by the enzyme. This method is adjusted according to Varon et al. [7].

#### Clinical examination and nerve conduction study

Clinical data were collected from ten children with CCFDN. All patients were examined by the authors. Clinical assessment included physical examination, neurological testing (PS, JH or DS), ophthalmological examination (AF), neurogenetic consultation (PS) and nerve conduction study (JH or DS).

## Results

### General results

All patients are of Roma Gypsy origin and presented a bilateral congenital cataract and microphthalmos and had early eye surgery on both eyes. Correct diagnosis was not made until the age of two years. All patients had variably delayed early motor milestones: independent gait was never achieved before the age of two.

**Figure 1:**
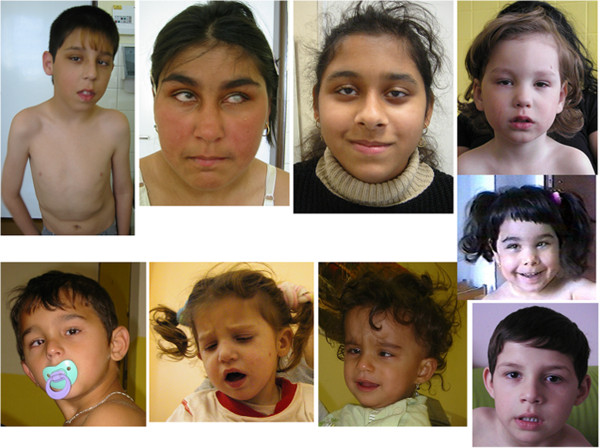
**Facial phenotype of Czech CCFDN patients.** Note that the facial dysmorphism (prominent nasal philtrum) is present rather in older children and that small children have quite normal appearance.

The facial features of the patients are presented in Figure 1.

### Neurological examination results

The gait is characteristically paleocerebellar in all the patients. Mental retardation was variable, but in most patients mild and they attend a special school. Three patients suffered an attack of rhabdomyolysis after a viral infection. One patient had already suffered four such attacks. The two oldest patients have had severe and progressive scoliosis since their teens and two have severe distal muscle atrophy and Achilles tendon shortening.

### Nerve conduction study results

Nerve conduction study showed severely decreased MNCVs to 20–35 m/s, slowing down with older age. The results are presented in Table 1.

**Table 1:** Clincal overview from ten Czech CCFDN patients

***Patient***	***Gender***	***Year at birth***	***Age at diagnosis and examination (years)***	***Congenital cataract surgery***	***Independent gait since the age of***	***Early motor milestones***	***Rhabdomyolysis***	***Scoliosis***	***Abnormal gait***	***Mental abilities***	***Neuropathy (MNCV)***
***1***	M	1993	11	Yes	Not before the age of 4 y.	Delayed	4x	No	Yes	Subnormal,special school	Demyelinating
											(22 m/s)
***2***	F	1991	14	Yes	4 y.	Delayed	1x	Yes	Yes	At 10 years as 3 years level -special school	Demyelinating
											(19–26 m/s)
***3***	M	1984	18	Yes	?	Delayed	No	Severe	unable	Subnormal	Demyelinating

***4***	M	2002	4	Yes	2.5	Delayed	No	No	Yes	Subnormal	Demyelinating
											(29 m/s)
***5***	M	2002	3.5	Yes	2 y.	Delayed	1x	No	Yes	Almost normal	Demyelinating

***6***	F	1990	14	Yes	2 y.	Delayed	No	No	Mildly	Borderline	Demyelinating
											(22 m/s)
***7***	F	2004	1.5	Yes	Not yet	Delayed	No	No	Unable	Subnormal	Demyelinating

***8***	F	2003	3	Yes	Not yet	Delayed	No	No	Unable	Subnormal	Demyelinating
											(33 m/s)
***9***	F	1996	4	Yes	?	Delayed	No	No	Unable	Subnormal	Demyelinating
***10***	M	2002	4.5	Yes	Not yet	Delayed	No	No	Unable	Subnormal	Demyelinating

### Ophthalmological examination results

All patients had dense congenital cataract and microphthalmos bilaterally. Horizontal corneal diameter varied between 7.5 mm and 9 mm at the time of diagnosis in infancy. All patients underwent cataract removal, either by extracapsular cataract extraction or by pars plicata lensectomy within the first year of life. Aphakic eyes were optically corrected by contact lenses after surgery. Six patients had nystagmus and final best-corrected visual acuity ranged between 0.02 and 0.5, in average 0.1 due to amblyopia. Lifelong follow-up in these patients is necessary to detect and treat late complication such as secondary glaucoma that was present in eight eyes of five patients at last follow-up visit.

### Photographs of the patients are presented in Figures 2, 3, 4, 5, 6, 7, 8, 9 and 10

**Figure 2:**
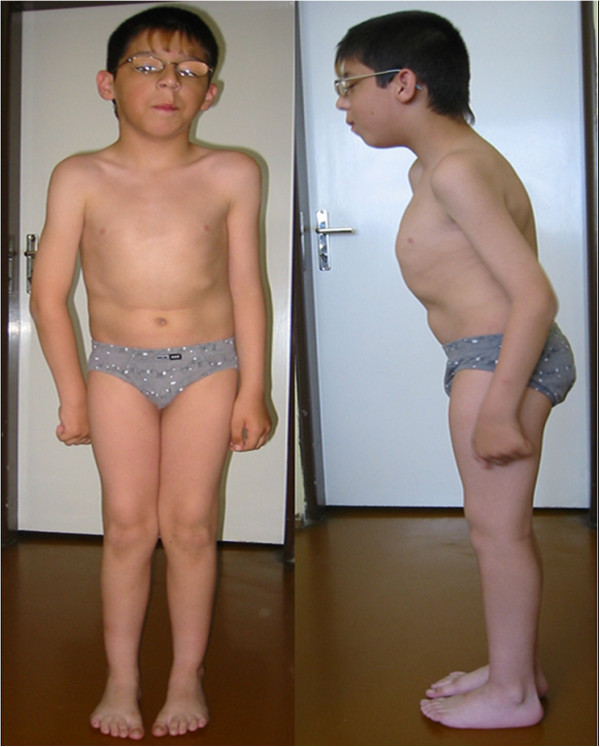
Patient 1, etc. Patient 1 at the age of 9 years (see text for details).

**Figure 3:**
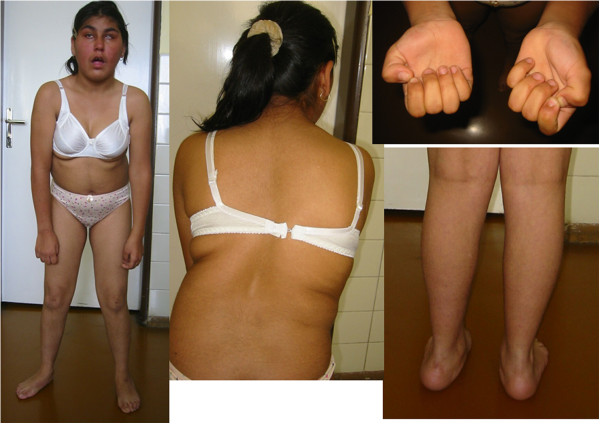
**Patient 2 at the age of 14 years.** Note the severe scoliosis and polyneuropathic signs of distal muscle weakness and atrophy. (see text for details)

**Figure 4:**
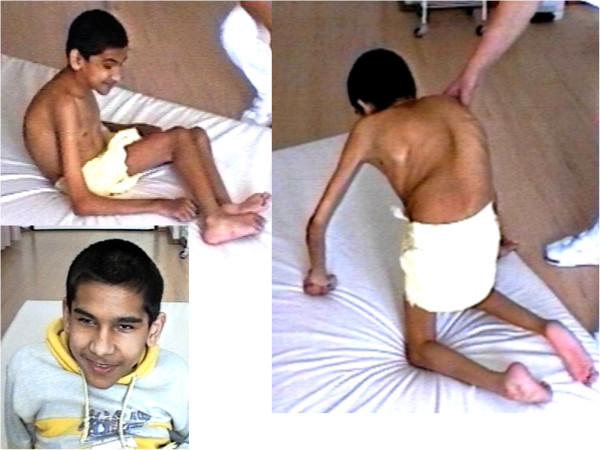
**Patient 3 at the age of 15.** This patient presented with dystrophic features (130 cm, 20 kg) and microcephaly (49 cm), facial dysfmorfism, microcornea and bilateral nystagmus. Note the severe contractures, scoliosis and pectus deformities and also the generalized muscle atrophy. He was not able to walk nor even stand [13].

**Figure 5:**
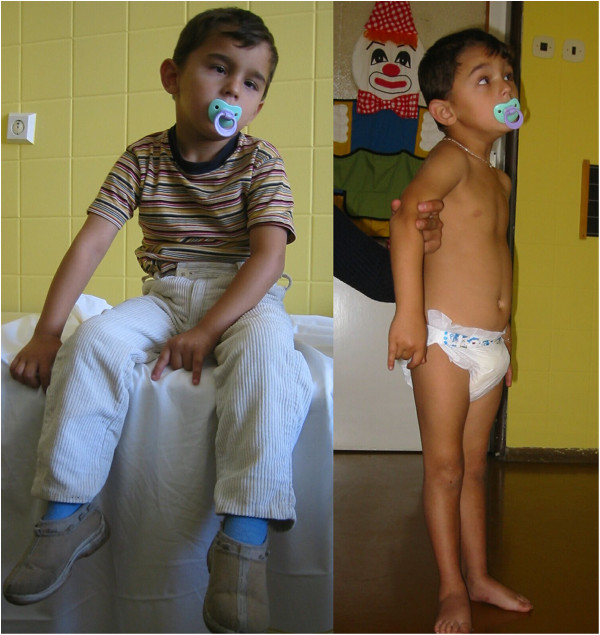
**Patient 4 at the age of 3.5 years.** The patient is a sporadic case in the family. He has no siblings. Prenatal screening revealed megaurether and hydronephrosis in this patient. Congenital cataract was surgically corrected at the age of three months. He was able to sit at 13 months of age and walk at 2.5 years. He is able to use simple sentences.

**Figure 6:**
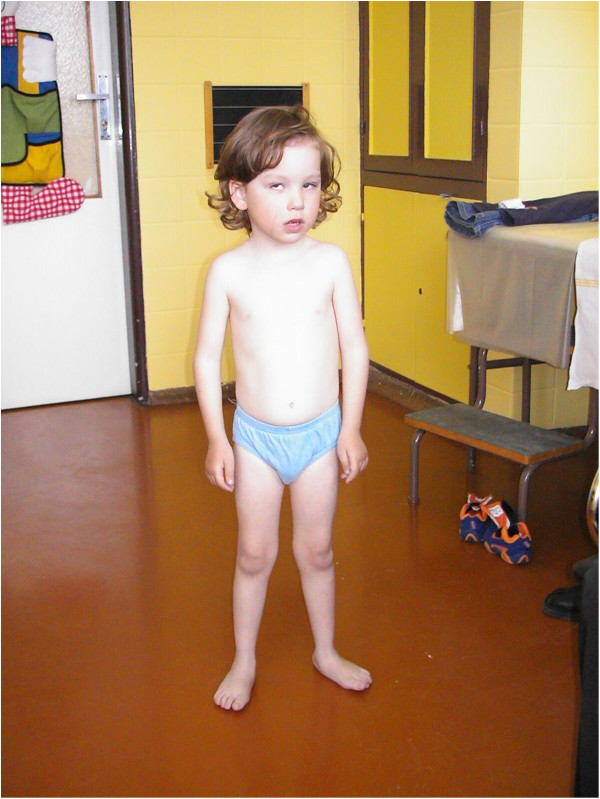
**Patient 5 at the age of 4.5 years.** The parents of the patient are consanquineous. They are second grade cousins. He had a rhabdomyolysis attack when he was two years old. He was misdiagnosed as Guillan-Barre syndrom. The proper diagnosis was made only when he was four years old, according to congenital cataract, developmental delay, demyelinating neuropathy and an attack of rhabdomyolysis.

**Figure 7:**
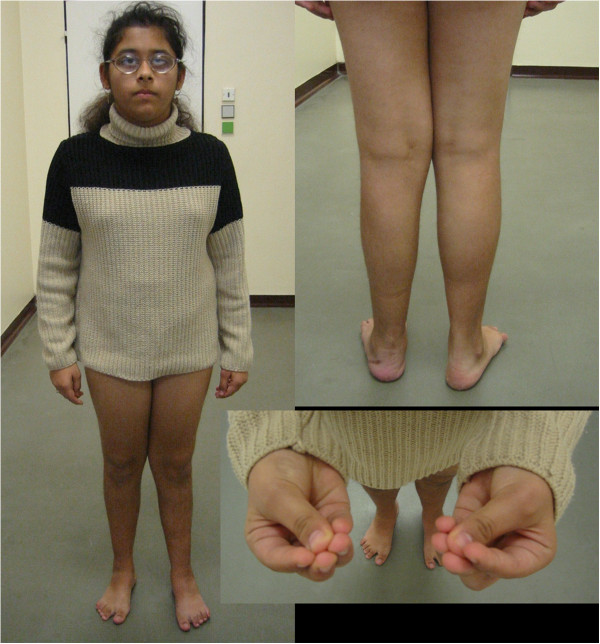
Patient 6 at the age of 17 years.

**Figure 8:**
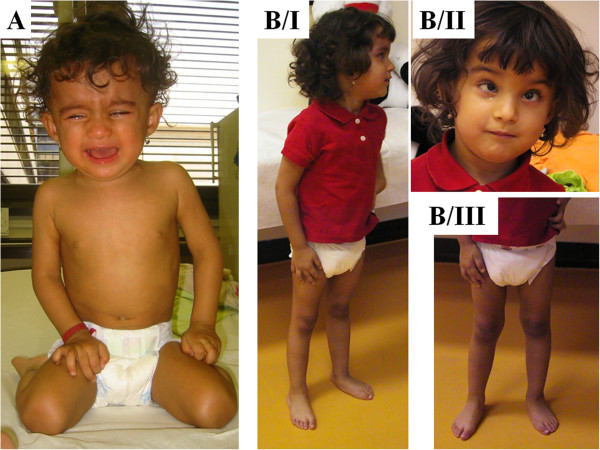
**Patient 7.** Panel **A**- at the age of 1.5 years. Panel **B**- at the age of 3.5 years. This girl is a sporadic case in the family; no other family members are affected. Panel **A**: The girl presented with developmental delay, she was able to sit at 12 months and to stand at 16 months of age, but she was not able to walk. Panel **B**: The girl presented with facial dysmorphism, demyelinating neuropathy and developmental delay.

**Figure 9:**
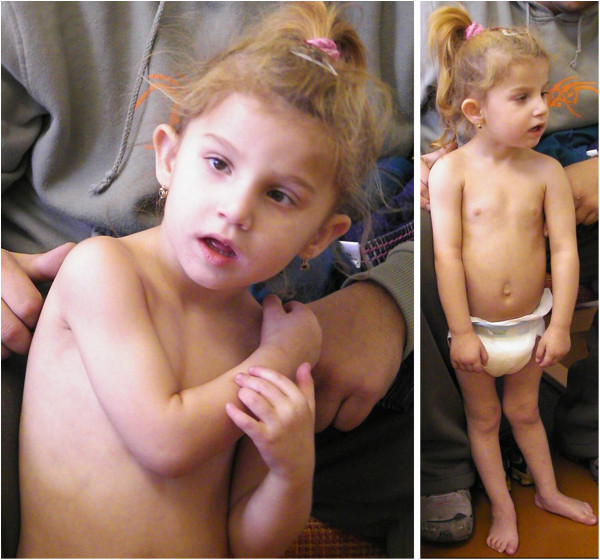
**Patient 8 at the age of 3.5 years.** The patient is a sporadic case in the family. At the age of 3.5 years she presented with facial dysmorphism, micropthalmus, demyelinating neuropathy and developmental delay. Congenital cataract was surgically corrected after birth. She was able to sit at 12 months of age, but is not able to walk.

**Figure 10:**
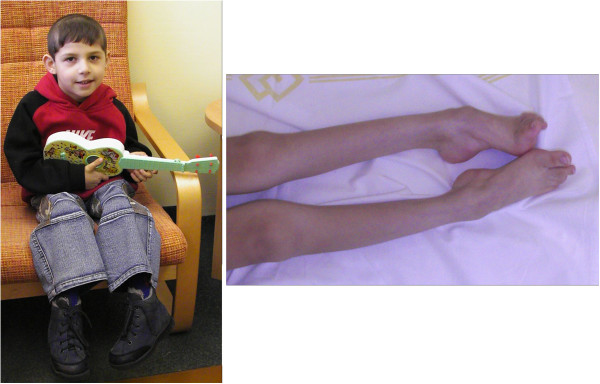
**Patient 10 at the age of 4.5 years.** The patient is a sporadic case in the family. At the age of 4.5 years he presented with pronounced distal muscle atrophies and early foot deformities.

#### Patient 1- Figure 2

This is a sporadic case in the family; the patient has two healthy brothers.

The bilateral congenital cataract was diagnosed soon after birth and surgical correction was performed.

The patient has been hospitalized several times at our department.

At the age of nine years he suffered from a first rhabdomyolysis attack after a viral infection. The patient was treated with forced diuresis. No dialysis was necessary. Serum creatine kinase and myoglobin levels, which were high initially, normalized after about 17 days. Myocardial or renal function abnormalities were not observed. The aetiology of the viral infection was not proved, however high levels of antibodies against Coxsackie and Adenomavirus were observed.

The second attack of rhabdomyolysis occurred at the age of 12 years. Serum creatine kinase levels after the second attack were (in μkal/l, normal levels are in range 0,19-2,27 μkal/l ): day 1: 679.00 – 300 x elevated; day 2: 542.00; day 4: 235.00; day 6: 150,00; day 9: 51.00; day 12: 20.27; day 18: 3.76.

Two other attacks of rhabdomyolysis followed when he was 15 and 16 years old and after both he recovered to his previous status.

#### Patient 2-Figure 3

This girl is a sporadic case in the family; no other family members are affected. Congenital cataract with microphthalmos was diagnosed soon after birth and later also a developmental delay was observed. Independent gait was achieved at the age of four years; she was able to speak in simple sentences when she was five years old. At the age of seven, she suffered an attack of rhabdomyolysis with initial serum creatine kinase level 627 μkat/l. Electromyography was done with the finding of acute myogenic lesion together with significantly reduced nerve motor and also sensory conduction velocities in peripheral nerves and therefore a diagnosis of peripheral demyelinating motor and sensory neuropathy was made [14].

When she was eight years old, she was hospitalized at our department. On neurological examination, mild mental retardation, muscle weakness and muscle atrophies were prominent. Hyporeflexia was noted. Scoliosis was present. Facial dysmorphism was observed.

The clinical course of the disease in other patients is summarized in Table 1 and in Figures 4, 5, 6, 7, 8, 9 and 10.

### Frequency of CCFDN among Czech patients of gypsy origin

Our data indicate for the high prevalence of CCFDN in the Czech Gypsy ethnic subpopulation. Our laboratory is the only laboratory offering and performing DNA testing for CCFDN in the Czech Republic. No other patients were diagnosed with CCFDN in other departments within the Czech Republic as far as we know. Since general screening for congenital cataract in new-borns was initiated in the Czech Republic in 2005, there is a good chance to diagnose all CCFDN patients early if all detected children of Gypsy origin with congenital cataract are tested for the c.863 + 389C > T mutation.

Among the 116 anonymous DNA samples from Czech and Slovak Gypsies we have not found any heterozygotes for the founder c.863 + 389C > T mutation.

Official data from the Czech Republic state that 4000 Gypsy children are born annually [15]. Literature-based estimates are that currently approx. 200 000 Roma (Gypsies) are living in the Czech Republic. (The estimates vary between 150–250 thousand) [16]. Overall, there are 10,5 million inhabitants in the Czech Republic, based on the data from the year 2013 [17].

We were able to collect a group of 10 patients with CCFDN. These patients were born in 1984, 1990, 1991, 1993, 1996, 2003 and 2004 respectively (one case per year) and three patients were born in 2002. The frequency of heterozygotes was calculated using simple Hardy-Weinberg equations.

During a period of 20 years, ten patients were diagnosed, therefore the incidence would be 10:80000 (4000*20). This is equivalent to an incidence of 1 newborn in 8000. Assortative mating is an significant phenomenon in this ethnic group.

## Discussion

We present a unique study of ten pediatric CCFDN patients of different ages. No similar study has been published before. Previous and original papers included adult patients and only a very few pediatric patients as case reports. Moreover, there have previously been reports about cohorts of CCFDN patients from Bulgaria, but only sporadic cases outside this country. We present probably the largest cohort of CCFDN patients except for the original report.

Some of our patients with CCFDN achieved independent gait only after the age of six years. However, the gait might be unstable. They are also capable of attending a school for mentally retarded. It is crucial to pay special attention to their needs because improvement of their skills is possible. Rehabilitation might be focused on prevention and treatment of scoliosis and feet deformities.

Patients with CCFDN are prone to rhabdomyolysis attack after viral infection. The exact cause of the attacks and its mechanism however remains unknown.

We tested 116 DNA control samples for the presence of the c.863 + 389C > T mutation. According to the Varon et al., we would expect a 0.6% (about 1 out of 167) carrier rate in general Gypsy population [7]. The mutation was not found in our control samples of limited size. While it is a very sensitive task to ask about the ethnic origin in the Czech Republic we were not able to collect more anonymized DNA control samples from the Gypsy population. Moreover, the control samples we had were collected in three distinct regions, all distant from each other (nothern Bohemia, Brno region and Slovakia) Therefore, these control individuals might not be as heterogenous as would be desirable. However, the observed frequency of heterozygotes is smaller than 1 in 116 (< 0.9%). Our data regarding the frequency of carriers of CCFDN are in accordance with Varon et al. CCFDN is a relatively common disorder in our Gypsy population. The situation might be the same also in other Central European countries where there is a majority of Romungo Gypsies (especially Hungary, Slovakia, the Czech Republic). Moreover, new migration waves after the 1989 also caused the spread of Romungo Gypsies to other European countries (Great Britain, Belgium, France, Spain, Germany) as well as non-European countries (Canada).

## Conclusions

Clinical diagnosis in CCFDN should be easy for an informed pediatrician or neurologist by the obligate signalling trias of bilateral congenital cataract, developmental delay and later demyelinating neuropathy. Motor NCVs decrease progressively with the patient’s age. All Gypsy patients with bilateral congenital cataract should be offered DNA testing for the CCFDN founder mutation. Newborn cataract screening should help to diagnose all CCFDN patients very early and provide them appropriate care and also genetic prevention to the family. Our data indicate a probably high prevalence of CCFDN in the Czech Gypsy ethnic subpopulation.

## Abbreviations

CCFDN: Congenital Cataract Facial Dysmorphism and demyelinating Neuropathy; CTPD1 gene: CTD phosphatase, unit 1; WWII: Second World War; PCR: Polymerase chain reaction.

## Competing interests

The authors report no competing interests.

## Authors’ contributions

PL and PS wrote the manuscript. JH, PS and DS performed clinical examination of the patients. JH and DS performed also the Nerve conduction studies. DS and PS collected the patients. AF performed the ophthalmological examination as well as cataract surgery in all patients. PS designed the study. All authors read, revised and approved the final version of the manuscript.
